# vWFpp/ADAMTS13 ratio is a useful marker of postliver transplantation thrombotic microangiopathy: A pediatric case report

**DOI:** 10.1002/ccr3.2495

**Published:** 2019-12-10

**Authors:** Lisa Duquenne, Samuel Balbeur, Emilie Everard, Raymond Reding, Stéphane Eeckhoudt, Bénédicte Brichard, Nathalie Godefroid, Emilien Derycke, Mina Komuta, Isabelle Scheers, Françoise Smets, Etienne Sokal, Xavier Stéphenne

**Affiliations:** ^1^ Division of Paediatric Gastroenterology and Hepatology Department of Paediatrics Cliniques Universitaires Saint Luc Université Catholique de Louvain Brussels Belgium; ^2^ Division of Paediatric Surgery Department of Surgery Cliniques Universitaires Saint Luc Université Catholique de Louvain Brussels Belgium; ^3^ Laboratory of Haematology Cliniques Universitaires Saint Luc Université Catholique de Louvain Brussels Belgium; ^4^ Division of Paediatric Haematology Department of Paediatrics Cliniques Universitaires Saint Luc Université Catholique de Louvain Brussels Belgium; ^5^ Division of Paediatric Nephrology Department of Paediatrics Cliniques Universitaires Saint Luc Université Catholique de Louvain Brussels Belgium; ^6^ Division of Emergency and Intensive Care Department of Paediatrics Cliniques Universitaires Saint Luc Université Catholique de Louvain Brussels Belgium; ^7^ Department of Anatomopathology Cliniques Universitaires Saint Luc Université Catholique de Louvain Brussels Belgium

**Keywords:** pediatric liver transplantation, sirolimus, tacrolimus, thrombotic microangiopathy, vWFpp/ADAMTS13 ratio

## Abstract

vWFpp/ADAMTS13 ratio should be further studied as a useful marker for diagnosis of thrombotic microangiopathy postliver transplantation. Immunosuppressive regimen modification and plasma supplementation can lead to recovery.

## Graphical abstract

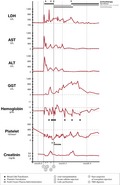

## 1 INTRODUCTION

Thrombotic microangiopathy (TMA) is a rare and life‐threatening complication following liver transplantation in children. Recent studies suggest that an elevated vWFpp/ADAMTS13 ratio could be a useful marker for diagnosis of thrombotic microangiopathy. This pediatric case report supports this hypothesis, and we propose, as treatment, immunosuppressive regimen modification and plasma supplementation***.***

Thrombotic microangiopathy is a microvascular occlusive disorder characterized by systemic or intrarenal aggregation of platelets leading to thrombocytopenia, mechanical destruction of red blood cells, and ischemic injury to the affected organs. This term includes a spectrum of diseases, all defined by the same histopathological finding: arteriolar and capillary thrombosis with specific abnormalities in the endothelium and vessel wall^1^, ^2^ Clinical presentation can be extremely variable. Diagnosis is generally based purely on biochemical findings in the presence of thrombocytopenia and nonimmune microangiopathic hemolytic anemia with schistocytes and negative direct Coombs test^2^

Thrombotic microangiopathy is a well‐recognized complication after renal and allogeneic hematopoietic stem cell transplantations. It is also increasingly reported following liver transplantation in adults.^2^, ^3^ Though endothelial damage seems a key event in all forms of TMA, so far, the exact pathophysiology of the disease is not completely understood and probably involves multiple mechanisms.^2^, ^3^, ^4^, ^5^ Early detection and aggressive treatment are vital to reduce significant morbidity and mortality associated with this disease. However, immediate accurate diagnosis is often difficult because of lack of rapid diagnosis test and so far, there is no standardized treatment protocol.^2^, ^3^, ^4^, ^5^

Recent studies suggest that a relative defect in ADAMTS13, a disintegrin and metalloprotease with thrombospondin type 1 domains, could contribute to the pathogenesis.^2^, ^6^, ^7^ We report a pediatric case of TMA following liver transplantation with increased von Willebrand factor pro‐peptide (vWFpp)/ADAMTS13 ratio at diagnosis, successfully treated with immunosuppressive regimen modification and plasma supplementation.

## 2 CASE PRESENTATION

The patient underwent an ABO‐compatible living‐related liver transplantation for genetically proven progressive familial intrahepatic cholestasis type 1 after failure of biliary diversion. She presented liver cirrhosis complicated by portal hypertension, failure to thrive, stunting and disabling pruritus. Post‐transplant immunosuppressive regimen consisted in steroid‐free induction with basiliximab (Simulect^R^, Novartis, Ixelles), and then tacrolimus (Prograft^R^, Astellas, Anderlecht) monotherapy, a calcineurin inhibitor (CNI). Tacrolimus blood levels were closely monitored, and drug doses were adjusted.

**Figure 1:**
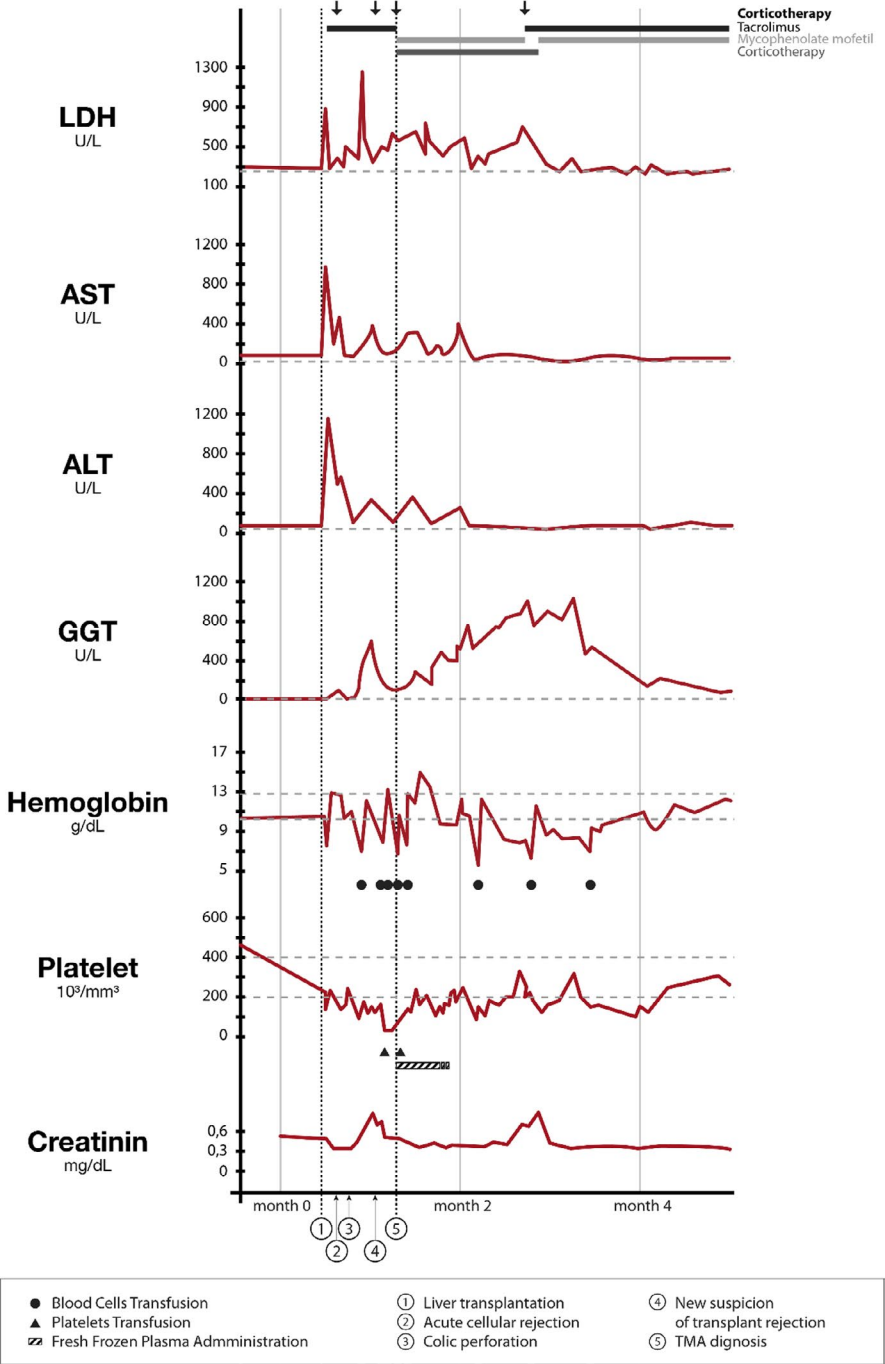
Trends in laboratory date in our patient. The patient had to stay in the pediatric intensive cares for 2 mo after TMA diagnosis. She faced lots of complications which explain the further transfusions and modification of renal function delayed from acute TMA, and not representing TMA relapse. We voluntary chose to not talk about those events in the presentation of this case report to avoid overload information

Five days after surgery, she developed acute cellular rejection with histologic confirmation. An echography was performed, showing no vascular trouble with permeable portal vein, sus‐hepatic veins, and hepatic artery. She was treated with intravenous methylprednisolone (Medrol^R^, Pfizer) (5 mg/kg/d for three days, then gradually tapered). Seven days postliving donor liver transplantation (LDLT), we observed normalization of transaminases. (Figure 1).

Nine days post‐transplant, colic perforation complicated by peritonitis was diagnosed. Surgical exploration revealed suture line dehiscence at the level of transverse colon. Segmental colonic resection trough laparotomy was performed, and adequate intravenous antibiotic therapy was administered.

At day 12 post‐transplantation, she developed acute respiratory distress syndrome linked to pleural effusion and diaphragmatic dyskinesia, requiring oxygen therapy and pleural drainage.

Fifteen days post‐transplant, blood tests revealed another increase in hepatic enzymes. As rejection was suspected, a new liver biopsy was performed, presenting a well‐delineated coagulation necrosis associated with canalicular damages and severe bilirubinostasis. She was treated with intravenous methylprednisolone bolus (10 mg/kg for 3 days and gradual tapering).

Despite normalization of hepatic function, the patient showed unexplained progressive deterioration of general condition, increased respiratory distress and increased transfusion requirements (transfusions of platelets and red blood cells), requiring a transfer to pediatric intensive cares.

Twenty‐one days post‐transplant, blood analysis revealed schistocytes at 11.5% with negative direct Coombs test. At this time, the patient also had low hemoglobin (6.4 g/dL) and platelet count (20 000/mm^4^), increased LDH (585 U/L), low haptoglobin (<0.1 g/dL), and elevated urea (87 mg/dL). ADAMTS13 levels were decreased at 11%, and WFpp/ADAMTS13 ratio was increased (48) (Table 1). Chest radiography showed signs of acute respiratory distress syndrome.

**Table 1:** Post‐transplant evolution of ADAMTS‐13 and von Willebrand factor pro‐peptide (vWfpp)

	vWFpp (%)^a^	ADAMTS‐13 (%)^a^	vWFpp/ADAMTS‐13^b^
Normal value	55‐229	>40%	3.66‐14.5
21 days post‐transplant	528 ↑	11 ↓	48 ↑↑
24 days post‐transplant	291 ↑	27 ↓	10.7
27 days post‐transplant	562 ↑	59	9.52
43 days post‐transplant	733 ↑	43	17 ↑
66 days post‐transplant	419 ↑	22 ↓	19 ↑

Note

↑, Values above normal range; ↓, Values below normal range.

a Normal values of Cliniques Universistaires Saint Luc's laboratory.

b Normal values after living donor liver transplantation extrapolated from the article of Takahashi N et al, 2013.^7^

Finally, thrombotic microangiopathy was diagnosed, with acute respiratory distress syndrome as complication.

Potential intercurrent bacterial, fungal, and viral infections were excluded. Unfortunately, no genetic test was performed to exclude other causes of primary TMAs.

We decided to change immunosuppression, and we switched to sirolimus (Rapamune^R^, Pfizer, Brussels) and mycophenolate mofetil (Cellcept^R^, Roche, Brussels) associated with methylprednisolone (5 mg/kg/dose). The patient also received fresh frozen plasma (FFP) once a day because of low ADAMTS13 blood levels, and multiple transfusions of platelets and packed red blood cells. We did not opt for therapeutic plasma exchange (as indicated in thrombotic thrombocytopenic purpura) because no autoantibodies against ADAMTS13 were detected in repeated laboratory tests.

Later on, blood parameters improved gradually and 10 days after treatment initiation, the frequency of FFP infusions was reduced and corticosteroid treatment was tapered. FFP administration was stopped 18 days after its initiation.

Eight weeks post‐transplant and 6 weeks after initially stopping tacrolimus, we observed normalized ADAMTS13 levels. Because of repeated pneumothorax and the potential impact of sirolimus on pleural scarring and because of persistent signs of cellular rejection on biopsies, we chose to restart tacrolimus and to stop sirolimus, while pursuing mycophenolate mofetil.

Finally, 19 weeks post‐transplant, the child's renal and hepatic functions had fully recovered. She was in good general condition and was discharged from hospital.

## 3 DISCUSSION

The case we presented supports the recent hypothesis that an increased vWFpp/ADAMTS13 ratio could be an early marker of postliver transplantation TMA (p‐LT TMA) in addition to the existing diagnostic criteria for TMA. Plasma supplementation and switch of immunosuppression (switch from CNIs‐based immunosuppressive regimen to sirolimus and mycophenolate mofetil) were proposed and were successful in this patient.

TMA is a rare, life‐threatening, and not fully understood complication after liver transplantation that needs prompt treatment.^8^, ^9^, ^10^, ^11^ However, the immediate accurate diagnosis of p‐LT TMA can be extremely difficult for multiples reasons: There is no current widely available rapid diagnostic test in suspected cases, patients can develop only minimal hematologic disorder or organ involvement, and differential diagnosis is required to distinguish p‐LT TMA from liver graft failure and disseminated intravascular coagulation.^3^, ^12^

It was the first case of TMA following living donor liver transplantation observed in the institution of Saint Luc in our cohort of more than 1000 operated children. This diagnosis was difficult because of its rareness, and the complexity of the multiple concomitant disorders encountered in this patient.

There are several distinct pathophysiologic mechanisms leading to TMA: ADAMTS13 deficiency‐mediated TMA, also called thrombotic thrombocytopenic purpura, defined by activity of ADAMTS13 below 5 to 10% (according to the authors) that can be congenital or due to the presence of an inhibitor or autoantibody (and so defined as acquired); Shiga toxins or *Streptococcus pneumoniae‐*related hemolytic‐uremic syndrome; alternative complement pathway dysregulation‐mediated TMA; drug‐mediated TMA; etc^1^, ^13^, ^14^ It is important to distinguish the etiologies in order to initiate appropriate treatment.^1^, ^13^

Underlying mechanisms of TMA remain largely unexplored. In the field of p‐LT TMA, different risk factors have been linked: ischemia‐reperfusion injury, immunosuppressive medications particularly CNIs (cyclosporine A and tacrolimus), interfering disease and relative ADAMTS13 deficiency.^2^

Calcineurin inhibitors administration as a risk factor is not a new concept^5^, ^15^, ^16^ but has recently been discussed.^17^ Their disease triggering effects have been linked to their ability to cause endothelial dysfunction resulting in vasoconstriction and increased platelet aggregation, possibly through the inhibition of prostacyclin.^2^, ^5^ However, CNIs are extensively used in transplanted patients and only a limited number of them will develop TMA, which can suggest the presence of another underlying factor.^17^ Likewise, CNIs withdrawal does not always guarantee a recovery.^2^, ^17^ mTOR inhibitors (as sirolimus) have also been reported to be implicated in the pathogenesis of TMA, probably due to their antiangiogenic properties.^2^ However, they are also used as rescue medication in cases of CNI‐induced TMA.^8^, ^16^, ^17^

Recent studies suggest that a relative defect in ADAMTS13 could contribute to the pathogenesis, more precisely an imbalance between ADAMTS13 and unusually large vWF multimers (UL‐vWFM).^2^, ^6^, ^7^ vWFpp is synthesized in endothelial cells and cleaved to form vWF. During endothelial damage, vWF is released into plasma as UL‐vWFM, the highly active form of vWF, which is essential for platelet aggregation at sites with a high degree of shear stress.^6^, ^18^ ADAMTS13 is a disintegrin and metalloprotease, which splits von Willebrand factor (vWF) multimers into small and inactive fragments, limiting platelet thrombus formation under normal conditions. Therefore, when ADAMTS13 activity is reduced, blood levels of UL‐vWFM increase and lead to excessive platelet clumping.^2^, ^6^, ^7^, ^18^, ^19^

ADAMTS13 deficiency seems to play a special role in TMA occurring after transplantation of the liver more than other solid organs.^2^, ^20^ Indeed, ADAMTS13 is synthesized in the liver. Consequently, graft dysfunction could lead to decrease its level which could lead to the fact that TMA tends to occur earlier after transplantation of the liver than other solid organs.^2^ Furthermore, we know that patients with cirrhosis have complex alterations in the hemostatic system. In particular, they have abnormally high plasma levels of vWF and low ADAMTS13 activity to compensate thrombocytopenia and thrombocytopathia^21^

Takahashi et al found that vWFpp/vWF ratio and vWFpp/ADAMTS13 ratio were significantly higher in the weeks following LDLT compared with pretransplant values and to healthy patients.^7^ They also highlighted that the vWFpp/vWF ratio was significantly higher in those who developed TMA, and the vWFpp/ADAMTS13 ratio significantly higher in nonsurvivors compared with survivors. These results suggest that a prothrombotic state exists in the recipients, and that pLT‐TMA would occur when the production of ADAMTS13 cannot meet the demand to cleave the oversynthesized UL‐vWFM released by the injured endothelium.^2^, ^6^, ^7^ Thus, recent studies suggest that a highly elevated vWFpp/ADAMTS13 ratio is a more useful marker, reflecting the prothrombotic state after LDLT, for the diagnosis of TMA and for poor outcome prediction compared with the independent values of ADAMTS13, vWF, and vWFpp^7^, ^11^, ^19^However, it should be noted that other studies showed some patients with p‐LT TMA had normal ADAMTS13 absolute values.^2^ Banno et al also proved with ADAMTS13‐deficient mice that complete deficiency in ADAMTS13 is not sufficient to cause a TMA. These authors suggest that other triggers, such as genetic defects or environmental factors (oxidative stress, complement dysfunction, and infection), are needed to provoke the disease.^22^ The frequent occurrence of p‐LT TMA concomitantly with other acute events such as infection or acute rejection (as in the situation of our patient) also supports this hypothesis.^2^, ^4^

In the case of our patient, ADAMTS 13’s value was superior to 10% and no antibodies were detected, excluding acquired thrombotic thrombocytopenic purpura. We found an increased vWFpp/ADAMTS‐13 ratio at the diagnosis, supporting its recent interest as a marker to identify TMA.

There is currently no available standard protocol for the treatment of post‐transplant TMA, probably because etiologies are multiple and clinical presentation is variable and heterogeneous. The treatment choice is controversial and can include supportive care, for example, transfusion of packed red blood cells and folic acid supplementation, replenishment of coagulation factors to substitute the deficient ADAMTS13 with plasma infusion/exchange and conversion or discontinuation of CNIs.^2^, ^4^

Although CNIs administration as main risk factor is recently discussed, the majority of authors agreed on modification of offending medication (CNIs discontinuation, dose reduction, or conversion) as first line of treatment.^2^, ^4^, ^17^ If possible, the CNI should be substituted by an immunosuppressive agent not linked with TMA, such as mycophenolate mofetil.^2^ However, clinicians must also consider the fact that CNIs modification may increase the risk of acute rejection.^2^

In this case, the switch for mycophenolate mofetil and sirolimus successfully addressed pLT‐TMA. However, because of secondary effects of sirolimus and persistent signs of cellular rejection, tacrolimus was reintroduced after TMA resolution. As the risk of developing TMA concerns especially the days following liver transplantation,^2^, ^20^ resuming tacrolimus was the option presenting the lowest risk. No signs of TMA relapse were observed.

A new medication is also studied in this indication: eculizumab, an anti‐C5 agent, that has already proven its efficacy as treatment and prevention of recurrent alternative complement pathway dysregulation‐mediated TMA. Its efficacy has already been documented in several cases of resistant medication‐associated TMA after renal transplantation with no evidence of genetic defects.^14^, ^17^ But this medication is currently extremely expensive, and some controversial results can be found in the literature.^14^, ^17^ Consequently, this option of treatment has to be further investigated and, in the meantime, has to be reserved to refractory patients.

Therapeutic plasma exchange is not indicated in the treatment of pLT‐TMA since no autoantibodies were detected.^2^, ^18^ Conversely, as we suppose that the pathogenesis of pLT‐TMA is linked to an imbalance between oversynthesized vWFpp and ADAMTS13 consumption, we consider the benefit of administration of plasma infusion to compensate a lack of ADAMTS13.

In this case, we finally managed to cure the patient from TMA after switching the immunosuppressive regimen to mycophenolate mofetil and sirolimus, and administering FFP.

## 4 CONCLUSION

After pediatric liver transplantation, it is necessary to consider the possibility of TMA, with its variable clinical presentations in view of the poor prognosis without early detection and aggressive treatment.^2^, ^4^, ^5^ The pathogenesis leading to the onset of this TMA is certainly multifactorial. Based on our experience and on previous studies, we suggest the vWFpp/ADAMTS13 ratio as an early marker to identify TMA in addition to the existing diagnostic criteria for TMA.

As a treatment, we propose switching immunosuppressive regimen to a non‐CNI and plasma supplementation to compensate insufficient production of ADAMTS 13.

However, further studies including larger cohorts of patients are necessary to confirm these approaches and to determine precise cutoff of vWFpp/ADAMTS13 ratio. Continuing investigations to increase understanding of the underlying physiopathology is essential to improve the prognosis and the care of these patients.

## AUTHOR CONTRIBUTION STATEMENTS

L. Duquenne: wrote the manuscript and validated the final section of the manuscript. X. Stéphenne: drafted initial sections of the manuscript, was in charge of the patient, and validated the final section of the manuscript. S. Balbeur: drafted initial sections of the manuscript and approved final section of the manuscript. E. Everard: drafted initial sections of the manuscript and approved final section of the manuscript. N. Godefroid: offered precious advices to write this manuscript and approved final section of the manuscript. B. Brichard: offered precious advices to write this manuscript and approved final section of the manuscript. É. Derycke: was in charge of the patient in the pediatric intensive care unit and validated the final section of the manuscript. R. Reding: served as a surgeon that operated the patient and validated the final section of the manuscript. S. Eeckhoudt: performed laboratory tests and validated the final section of the manuscript. É. Sokal, F. Smets, and I. Scheers: were in charge of the patient and validated the final section of the manuscript. All authors: provided critical feedback and helped shape the manuscript.

## CONFLICT OF INTEREST

None.

## INFORMED CONSENT STATEMENT

Informed written consent was obtained from the patient for publication of this report and any accompanying images.

## References

[1] George JN , Nester CM . Syndromes of thrombotic microangiopathy. New Engl J Med. 2014;371(7):654‐666. 25119611 10.1056/NEJMra1312353

[2] Verbiest A , Pirenne J , Dierickx D . De novo thrombotic microangiopathy after non‐renal solid organ transplantation. Blood Rev. 2014;28(6):269‐279. 25266355 10.1016/j.blre.2014.09.001

[3] Nishi H , Hanafusa N , Kondo Y , et al. Clinical outcome of thrombotic microangiopathy after living‐donor liver transplantation treated with plasma exchange therapy. Clin J Am Soc Nephrol. 2006;1(4):811‐819. 17699291 10.2215/CJN.01781105

[4] Shindoh J , Sugawara Y , Akamatsub N , et al. Thrombotic microangiopathy after living‐donor liver transplantation. Am J Transplant. 2012;12(3):728‐736. 22070669 10.1111/j.1600-6143.2011.03841.x

[5] Nwaba A , MacQuillan G , Adams LA , et al. Tacrolimus‐induced thrombotic microangiopathy in orthotopic liver transplant patients: case series of four patients. Int Med J. 2013;43(3):328‐333. 10.1111/imj.12048 23441660

[6] Nakanuma S , Miyashita T , Hayashi H , et al. Von Willebrand Factor deposition and ADAMTS‐13 consumption in allograft tissue of thrombotic microangiopathy‐like disorder after living donor liver transplantation: A case report. Transplantation Proceeding. 2017;49(7):1596‐1603. 10.1016/j.transproceed.2017.02.047 28651806

[7] Takahashi N , Wada H , Usui M , et al. Behavior of ADAMTS13 and Von Willebrand factor levels in patients after living donor liver transplantation. Thromb Res. 2013;131:225‐229. 23266519 10.1016/j.thromres.2012.12.002

[8] Czubkowski P , Pawłowska J , Jankowska I , et al. Successful sirolimus rescue in tacrolimus‐induced thrombotic microangiopathy after living‐related liver transplantation. Pediatr Transplant. 2012;16(6):E261‐E264. 22066835 10.1111/j.1399-3046.2011.01601.x

[9] Lee CH , Chen CL , Lin CC , et al. Plasma exchange therapy for thrombotic thrombocytopenic purpura in pediatric patients with liver transplantation. Transplant Proceed. 2008;40(8):2554‐2556. 10.1016/j.transproceed.2008.07.011 18929799

[10] Takatsuki M , Uemoto S , Kurokawa T , Koshiba T , Inomata Y , Tanaka K . Idiopathic thrombocytopenic purpura after a living‐related liver transplantation. Transplantation. 1999;67:479‐481. 10030298 10.1097/00007890-199902150-00023

[11] Hori T , Kaido T , Oike F , et al. Thrombotic microangiopathy‐like disorder after living‐donor liver transplantation: A single‐center experience in Japan. World J Gastroenterol. 2011;17(14):1848‐1857. 21528059 10.3748/wjg.v17.i14.1848 PMC3080720

[12] Plautz WE , Raval JS , Dyer MR , Rollins‐Raval MA , Zuckerbraun BS , Neal MD . ADAMTS13: origins, applications, and prospects. Transfusion. 2018;58(10):2453‐2462. 30208220 10.1111/trf.14804

[13] Kottke‐Marchant K . Diagnostic approach to microangiopathic hemolytic disorders. Int J Lab Hematol. 2017;1:69‐75. 10.1111/ijlh.12671 28447417

[14] Loirat C , Rémeaux‐Bacchi V . Atypical uremic hemolytic syndrome. Orphanet Journal of Rare Diseases. 2011;6:60. 21902819 10.1186/1750-1172-6-60 PMC3198674

[15] Zarifian A , Meleg‐Smith S , O’Donovan R , Tesi RJ , Batuman V . Cyclosporine‐associated thrombotic microangiopathy in renal allografts. Kidney Int. 1999;5:2457‐2466. 10.1046/j.1523-1755.1999.00492.x 10354295

[16] Fortin MC , Raymond MA , Madore F , et al. Increased risk of thrombotic microangiopathy in patients receiving a cyclosporin‐sirolimus combination. Am J Transplant. 2004;4(6):946‐952. 15147429 10.1111/j.1600-6143.2004.00428.x

[17] Abbas F , Kossi ME , Kim JJ , Sharma A , Halawa A . Thrombotic microangiopathy after renal transplantation: current insights in de novo and recurrent disease. World J Transplantation. 2018;8(5): 122‐141. 10.5500/wjt.v8.i5.122 PMC6134269 30211021

[18] Long ZX . ADAMTS13 and von willebrand factor in thrombotic thrombocytopenic purpura. Ann Rev Med. 2015;66(1):211‐225. 25587650 10.1146/annurev-med-061813-013241 PMC4599565

[19] Akyol O , Akyol S , Chen CH . Update on ADAMTS13 and VWF in cardiac and hematological disorders. Clin Chim Acta. 2016;463:109‐118. 27746209 10.1016/j.cca.2016.10.017

[20] Ko S , Okano E , Kanehiro H , et al. Plasma ADAMTS13 activity may predict early adverse events in living donor liver transplantation: observations in 3 cases. Liver transplant. 2006;12(5):859‐869. 10.1002/lt.20733 16528712

[21] Lisman T , Bongers TN , Adelmeijer J , et al. Elevated levels of von Willebrand Factor in cirrhosis support platelet adhesion despite reduced functional capacity. Hepatology. 2006;44(1):53‐31. 16799972 10.1002/hep.21231

[22] Banno F , Kokame K , Okuda T , et al. Complete deficiency in ADAMTS13 is prothrombotic, but it alone is not sufficient to cause thrombotic thrombocytopenic purpura. Blood. 2006;107(8):3161‐3166. 16368888 10.1182/blood-2005-07-2765

